# Equations for Assessing Body Composition by Ultrasound in Older Adults: A Narrative Review

**DOI:** 10.3390/healthcare13111295

**Published:** 2025-05-29

**Authors:** Lara Vilar Fernandes, Gabriela Benatti de Oliveira, Ana Carolina Junqueira Vasques, Ligiana Pires Corona

**Affiliations:** 1 School of Applied Sciences, State University of Campinas, Limeira 13484-350, Brazil; ana.vasques@fca.unicamp.br; 2 School of Medical Sciences, State University of Campinas, Campinas 13083-887, Brazil; g235166@dac.unicamp.br

**Keywords:** aging, body composition, muscle mass, body fat, ultrasound

## Abstract

As individuals age, physiological changes in body composition occur, potentially contributing to adverse health outcomes in the elderly population. Various methods are used to assess body composition, but gold-standard techniques often involve technical complexity, high costs, and lack of portability. Alternative methods that are portable, relatively low-cost, and technically simpler are necessary for clinical use. Due to its portability, safety, and lower cost compared with gold-standard equipment, B-mode ultrasound has been suggested as a potential method for body composition assessment. This narrative review aimed to identify and discuss equations developed using ultrasound to assess body composition in older adults. An electronic search was conducted in the MEDLINE/PubMed, Web of Science, Scopus, and Google Scholar databases in September 2023 and updated in April 2025. The search terms used were ultrasound, body composition, muscle, fat, older adults, aging, and equation. To date, no standardized cut-off points have been established to define low muscle mass or a high body fat percentage using ultrasound in older adults. Further research is needed to determine the validity and applicability of this technique in comparison with gold standard methods, as well as among the different types of ultrasound devices (A-mode and B-mode). Caution is warranted when selecting predictive equations for assessing body composition in clinical practice in older adults, as several factors related to equipment and the population assessed must be taken into account.

## 1. Introduction

Aging is associated with progressive changes in body composition, including reductions in lean mass and increased fat accumulation, especially in the visceral regions [1,2,3]. These changes can contribute to reduced food intake during aging and, consequently, to the development of unfavorable outcomes related to nutritional deficiencies, such as sarcopenia, frailty, and malnutrition.

A review study reported an approximate decline of 4.7% in muscle mass per decade in men and 3.7% in women [4]. A literature review that included studies from several countries observed a prevalence of sarcopenia in community-dwelling older adults of 17% according to EWGSOP1 and 11% according to EWGSOP2, reporting a range from 6.2% to 35.3% for EWGSOP1 and 3.2% to 26.3% for EWGSOP2 [5]. Skeletal muscle performs several essential functions in the human body, not only being related to mobility and force production [6], and is considered a secondary secretory organ with endocrine functions [7]. A greater muscle mass has a protective association with cardiovascular and all-cause mortality in older adults [8]. Wang et al. (2023) highlight the importance of preventing low muscle mass to contribute to reducing mortality risk and promoting healthy longevity [9].

Adipose tissue is a regulator of systemic metabolism and body homeostasis [10]. In older adults, there is a significant difference in fat distribution between men and women, with men tending to accumulate more central and visceral adipose tissue and being more likely to deposit visceral fat in the abdominal region. In contrast, women tend to have more peripheral subcutaneous adipose tissue and are more likely to deposit fat in the lower extremities [11]. Increased body fat contributes to adipocyte dysfunction through increased production of pro-inflammatory cytokines and insulin resistance, which may contribute to the development of metabolic and cardiovascular diseases in older adults [10]. According to the commission that defined new diagnostic criteria for obesity published by The Lancet, obesity is often diagnosed by body mass index, but the approach should be more precise, incorporating measurements of body fat and clinical signs of health [12].

The body composition changes associated with aging are influenced not only by the physiological aging process but also by modifiable factors such as diet, physical inactivity, and chronic diseases [13]. They may negatively impact health, physical function, and quality of life in older adults [1,2].

Understanding the dynamics of these alterations and their clinical consequences is essential for informing prevention strategies and interventions aimed at promoting healthy aging in diverse older populations. Therefore, it is critically important to accurately assess body composition to support the prevention and treatment of nutrition-related diseases.

Various tools are available for assessing muscle mass and body fat in populations. Literature reviews have outlined the advantages and disadvantages of each method, such as computed tomography (CT), dual-energy X-ray absorptiometry (DXA), bioelectrical impedance analysis (BIA), calf circumference (CC), and ultrasound (US) [14,15]. CT and DXA are considered gold standard methods, but CT involves radiation exposure and lacks standardized reference values. Both CT and DXA are expensive, non-portable, size-restrictive (height and weight), and require trained technicians.

Although BIA is more affordable and portable than CT and DXA, it requires population-specific equations based on age and ethnicity. The results may also be influenced by hydration status, fasting, and recent physical activity, potentially limiting their use in older adults. Calf circumference assessment may be confounded by body mass index (BMI) and peripheral edema and may not detect small muscle mass changes over short follow-up periods [16]. Thus, CC is recommended only in settings where other equipment is unavailable [17].

It is interesting to note that some studies have also demonstrated the possibility of using machine learning to assess body composition, but they are still little explored and will not be our focus in this work. One example is the study by Jeon et al. (2023), who proposed a machine learning-based obesity classification model using anthropometric measurements obtained by 3D body scanning to overcome the limitations of BMI in estimating adiposity [18]. Compared with BMI and BIA, the model demonstrated greater accuracy in classifying obesity when validated against DXA. Despite the promising results, this study has important limitations, such as the use of a sample restricted to young Korean adults, small sample size, and low accessibility to 3D scanning equipment [18]. These findings reinforce the potential of alternative technologies, such as ultrasound, in assessing body composition, which stands out for its portability, lower cost, and broader clinical applicability.

In contrast, ultrasound is a clinical imaging tool that has been used for decades. Some equations have been developed to estimate body composition using ultrasound [19,20,21]. However, studies on this topic remain limited, especially among older adults, an important population for body composition assessment due to age-related health risks such as sarcopenia, frailty, falls, disability, and premature mortality [1,2,22].

Therefore, portable, cost-effective, and low-complexity methods are essential for clinical practice. Given its portability, safety, and lower cost compared with gold-standard devices, B-mode ultrasound has been proposed as a practical method for assessing body composition in research, clinical, and hospital settings [23,24].

In recent years, growing evidence has highlighted the potential of ultrasound as a valuable tool for both nutritional assessment and guidance of nutritional interventions [25]. Ultrasound can assess key components of the Global Leadership Initiative on Malnutrition (GLIM) criteria for malnutrition, including low body mass index [26], unintentional weight loss [26], reduced skeletal muscle mass [27,28], and impaired food intake or assimilation [29], thereby supporting both phenotypic and etiologic diagnoses [30]. In addition, a certain degree of examiner proficiency is necessary, and inter-rater reliability has sometimes been questioned, even though larger muscles, like the quadriceps, are generally well visualized. Consequently, the use of ultrasound by registered dietitians presents specific practical challenges [25].

In September 2023, the Brazilian Federal Council of Nutritionists (CFN) issued Technical Note No. 83/2023 [31] regarding the use of ultrasound for body composition assessment by nutritionists. It highlighted that only one portable device has been specifically developed for this purpose. However, the CFN later removed the note from its website and social media, likely due to negative reactions from the professionals. Consequently, it is crucial to better understand the applicability of ultrasound and the types of equipment used to validate equations and ensure proper handling by health professionals.

Despite the growing interest in using ultrasound to assess body composition in older adults, there remains limited consensus regarding the validity and applicability of existing predictive equations. Many were originally derived from younger or athletic populations, raising concerns about their accuracy in older adults, who often present with age-related changes in body composition, such as increased fat infiltration in the muscles. Moreover, differences in ultrasound mode (A-mode and B-mode), measurement protocols, and population characteristics hinder direct comparisons and broad clinical applications.

Although previous reviews have examined the use of ultrasound to assess the muscle or fat components of body composition, they have generally focused on broader methodological aspects, such as measurement reliability and anatomical landmarks [32,33,34]. Therefore, a narrative review of the predictive equations available in the literature is essential to identify their strengths, limitations, and suitability for use in older adults.

In this context, the objective of the present study was to review and discuss predictive equations developed using measurements of muscle and subcutaneous fat thickness via A-mode and B-mode ultrasound to assess body composition in older adults.

## 2. Materials and Methods

The electronic search for articles included in this narrative review was conducted in the following databases: MEDLINE/PubMed, Web of Science, Scopus, and Google Scholar, initially in September 2023 and updated in April 2025 by a single researcher (L.V.F.). The search terms were combined using Boolean operators “AND” and “OR”: [“ultrasound”, “body composition”, “muscle”, “fat”, “aging”, “older adults”, “equation”].

Original studies published in English and Portuguese were selected, with publication dates limited to between 2014 and 2025. The decision to limit the literature review to studies from 2014 to 2025 was based on the need to capture up-to-date research. Over the past decade, significant advancements have been made in portable ultrasound devices, image resolution, software-assisted measurements, and standardized protocols, particularly in studies focusing on older adults. Moreover, interest in sarcopenia, frailty, and age-related body composition changes has increased considerably during this period, leading to a surge in research specific to older populations. Earlier studies, while foundational, often focused on younger or athletic populations. The review included studies that developed predictive equations for assessing body composition (muscle mass or body fat) using ultrasound in healthy older adults. The term “healthy” was defined according to the methodologies used in the included studies.

This narrative review was conducted following the SANRA scale (Scale for the Assessment of Narrative Review Articles) [35].

## 3. Results and Discussion

### 3.1. Ultrasound as a Method for Body Composition Assessment

Although aging naturally leads to significant changes in body composition, the loss of muscle mass can be accelerated by various factors, such as physical inactivity, reduced nutrient intake and absorption, and systemic inflammation [16]. Declining muscle health and malnutrition are common in older adults and contribute to functional impairment, reduced quality of life, loss of autonomy, falls and fractures, physical disability, disease progression, and decreased survival [16].

In this context, body composition assessment is a routinely used tool to detect or diagnose nutrition-related conditions, such as malnutrition, overweight, obesity, sarcopenia, and sarcopenic obesity, all of which have significant public health implications. Furthermore, body composition data provide essential guidance for selecting the most appropriate treatment for each patient. Understanding the most accurate techniques and protocols for assessing body composition enables healthcare professionals to effectively perform these functions in clinical settings [36].

Ultrasound is a low- to moderate-cost imaging method that does not involve radiation exposure. It is widely used to assess muscle alterations and, in many cases, offers the advantage of portability, allowing assessments to be conducted in different settings. Additionally, ultrasound provides real-time imaging with short acquisition times [23].

Clinical ultrasound operates in two imaging modes: amplitude modulation (A-mode) and brightness modulation (B-mode). A-mode displays a peak in the ultrasound wave amplitude at the interface between two distinct tissues, such as the subcutaneous fat and muscle. In contrast, B-mode generates two-dimensional images of underlying tissues [37]. B-mode ultrasound has broader clinical applications and is more commonly used than A-mode; however, B-mode devices are typically larger, more expensive, and require greater expertise for accurate image interpretation [37].

### 3.2. Ultrasound for Muscle Mass Assessment in Older Adults

B-mode ultrasound has been proposed as a suitable tool for estimating both the quantity and quality of muscle tissue [38]. Muscle thickness measured using B-mode ultrasound has shown a strong correlation with muscle mass assessed by DXA in both adults and older adults and has, therefore, been used in predictive equations to estimate muscle mass [19,38]. Moreover, studies evaluating the validity of these equations are important for assessing their clinical accuracy [24].

B-mode ultrasound has been employed to estimate muscle mass for diagnosing sarcopenia in older adults. A study involving 103 older adults (57 men and 46 women) at risk of sarcopenia developed algorithms using muscle thickness measurements at multiple anatomical sites. Muscle thickness at Site 7 (rectus femoris and vastus intermedius) showed the strongest correlation with the skeletal muscle mass index in males (R = 0.719, *p* < 0.001). In females, the highest correlation was observed at Site 3 (flexor pollicis longus, flexor digitorum superficialis, and brachioradialis), with R = 0.733 (*p* < 0.001). Site 7 was selected for the single-site prediction model, yielding an R^2^ of 0.701 and a standard error of estimate (SEE) of 0.519 kg/m^2^. The four-site algorithm demonstrated high accuracy (R^2^ = 0.819) and a low standard error of the estimate (SEE = 0.404 kg/m^2^), suggesting that this tool may support the diagnosis of sarcopenia [39].

Although previous studies [40,41] found abdominal or calf muscle thickness to be strong indicators of sarcopenia or low skeletal muscle mass, the results of this review suggest that the quadriceps may be more accurate for estimating muscle mass. This difference may be related to age-related atrophy patterns, as calf muscle loss may not follow a linear decline in the skeletal muscle mass index. Additionally, variations in scanning protocols, such as participant positioning, may have contributed to these divergent findings [39]. However, each muscle group offers distinct clinical value given its specific function [39].

B-mode ultrasound is a non-invasive and accessible method for estimating muscle thickness in older adults and shows a good correlation with DXA-derived lean mass, particularly with forearm measurements [42]. One study showed that ultrasound has good validity for assessing muscle size in older adults compared to DXA and CT, supporting its use in clinical practice. However, its reliability for evaluating small muscles requires further investigation, and the cross-validation of predictive equations in older populations is essential before it can be considered a valid alternative for diagnosing sarcopenia [32].

Nijholt et al. (2017) [32] reviewed 17 studies on the reliability and validity of ultrasound for assessing muscle quantity in older adults, reporting intra-class correlation coefficient (ICC) values ranging from −0.26 to 1.00; higher reliability (ICC = 0.72–1.00) was observed for measurements of the vastus lateralis, rectus femoris, upper anterior arm, and trunk muscles. All validity studies reported excellent reliability, with ICC between 0.92 and 0.99. Additionally, two studies demonstrated a strong agreement between ultrasound and DXA for estimating lean body mass, with R^2^ values ranging from 0.92 to 0.96. This systematic review [32] supports the use of ultrasound as a reliable method for assessing muscle size in older adults. However, further high-quality studies are required to confirm these findings across diverse populations. Additional research is warranted to evaluate its use in small muscles and to validate lean-mass prediction equations in older adults with different health and functional statuses [32].

Table 1 presents the methodologies of studies involving older adults in which predictive equations for muscle mass assessment were developed using B-mode ultrasound.

The standard error of the estimate (SEE), as shown in Table 2, reflects the uncertainty related to the mean population estimate and indicates the precision of the predictive parameters. A lower SEE is desirable and typically improves with larger sample sizes [43]. Um valor de SEE < 3% é considerado como muito bom [44]. Values below 3% were considered highly accurate [44]. The coefficient of determination (R^2^), which ranges from 0 to 1, measures the strength of the linear relationship between the variables. Values closer to 1 indicate a stronger association [45].

**Table 1:** Methodological description of studies that developed predictive equations to estimate muscle mass using B-mode ultrasound in older adults.

Author, Year	Ultrasound Type	Reference Method	Sample Size	Population	Ethnicity	Sex	Age (Years)
Abe et al., 2015 [46]	B-mode (Aloka SSD-500)	DXA	Development: 71/Cross-validation: 31	Healthy adults and older adults	Caucasian	M & F	50–76
Abe et al., 2018 [47]	B-mode (Aloka SSD-500)	DXA	Development: 389/Bootstrap validation: 1000 replications	Healthy older adults	Asian	M & F	60–79
Barbosa-Silva et al., 2021 [19]	B-mode (Xario SSA-660A)	DXA	Development: 190/Bootstrap validation: 10,000 replications	Healthy older adults	Caucasian	M & F	60–90
Takai et al., 2014 [21]	B-mode (Aloka SSD-900)	DXA	Development: 77	Healthy adults and older adults	Asian	M & F	52–78
Yuguchi et al., 2022 [20]	B-mode (Minato Medical Science Co.)	BIA	Development: 193	Healthy older adults	Asian	M & F	≥65
Paris et al., 2017 [38]	B-mode (M-Turbo, SonoSite)	DXA	Development: 96/Cross-validation: 96	Healthy adults and older adults	Caucasian	M & F	24–72

Note. DXA = Dual-energy X-ray absorptiometry; BIA = Bioelectrical Impedance Analysis; M = male; F = female.

Table 2 shows the predictive equations developed to estimate muscle mass in older adults using B-mode ultrasound as reported in the literature.

**Table 2:** Predictive equations were developed to estimate muscle mass using B-mode ultrasound in older adults.

Author, Year	Equation Outcome	No. of Parameters	Equation	SEE (kg)	R^2^	Adjusted R^2^
Abe et al., 2015 [46]	ALM	5	ALM (kg) = 4.32 × MT-FA (cm) + 2.98 × MT-UA (cm) + 2.85 × MT-LA (cm) + 0.97 × MT-TP (cm) + 0.94 × MT-LP (cm) − 23.12	1.5	0.9	0.94
		7	ALM (kg) = 3.67 × MT-FA (cm) + 2.84 × MT-UA + 2.58 × MT-LA + 1.05 × MT-TP + 0.93 × MT-LP + 0.069 × age + 0.79 × MT-TA − 27.63	1.4	0.9	0.95
Abe et al., 2018 [47]	ALM	4	ALM = −2.0940 + (sex × 4.1273) − (age × 0.0094) + (MT-FA × height × 3.5699) − (sex × age × 0.0307) − (sex × MT-FA × height × 0.8349)	—	0.8	0.86
		7	ALM = −7.9116 + (sex × 5.1693) + (age × 0.0345) + (MT forearm anterior × height × 2.2752) + (MT-UA × height × 0.0743) + (MT-TA × height × 0.4927) + (MT-LA × height × 1.4892) − (sex × age × 0.0380) − (sex × MT-FA × height × 0.3379) − (sex × MT-UA × height × 0.1263) − (sex × MT-TA × height × 0.1754) − (sex × MT-LA × height × 0.3083)	—	0.9	0.89
Barbosa-Silva et al., 2021 [19]	AMM	7	ALM = 3.27 × sex (0 = F, 1 = M) + 16 × height (m) + 0.2 × arm length (cm) + 0.09 × dominant arm circumference (cm) + 0.04 × dominant thigh circumference (cm) + 1.25 × dominant arm MT (cm) + 0.72 × dominant thigh MT (cm) − 24.9	1.23	-	0.90
		5	ALM = 2.39 × sex + 15.14 × height (m) + 0.29 × arm length (cm) + 1.93 × dominant arm MT + 0.87 × dominant thigh MT − 23.78	1.3	-	0.89
Takai et al., 2014 [21]	FFM	5	FFM = (sex × 7.217) + (MT-TA × 1.985) + (MT-TP × 2.355) + (MT-LA × 3.633) + (MT-LP × 2.670) − 6.759	2.5	0.9	—
		5	FFM = (sex × 5.233) + (MT × upper arm anterior length × 0.006630) + (MT × thigh anterior length × 0.05153) + (MT × thigh posterior length × 0.05579) + (MT × lower leg posterior length × 0.07097) + 1.774	2.0	0.9	—
Yuguchi et al., 2022 [20]	SMMI	3	SMI = 1.27 × sex + 0.18 × BMI + 0.09 × MT gastrocnemius (mm) + 1.3	—	0.8	0.80
Paris et al., 2017 [38]	AMM	7	ALM = 2.929 + 1.555 × (five-site MT × height) − 1.985 × sex (male = 0; female = 1) + 0.0247 × age	1.6	—	0.91

Note. ALM = Appendicular lean mass; MT-FA = forearm; MT-UA = upper-arm anterior; MT-LA = lower-leg anterior; MT-TP = thigh posterior; MT-LP = lower-leg posterior; MT-TA = thigh anterior; FFM = Fat-free mass; SMI = Skeletal muscle mass index; MT = Muscle thickness; BMI = Body mass index; Sex: 0 = female, 1 = male; SEE = standard error of the estimate.

The equations developed by Abe et al. (2015) estimated appendicular muscle mass with high precision and relatively low error compared to DXA measurements. In the model-development group (n = 71), significant correlations (*p* < 0.05) were found between DXA-derived appendicular lean mass and ultrasound-measured muscle thickness at 10 sites (r = 0.379–0.936). Prediction errors ranged from 1.78 to 2.54 kg for model series A and from 1.53 to 2.23 kg for model series B. Bland-Altman analysis showed no systematic bias in appendicular lean mass and predictions for the validation group. These equations incorporate muscle thickness from the limbs, as the DXA-derived appendicular mass does not include trunk musculature. Since muscle mass loss with aging tends to be more pronounced in the lower limbs, the inclusion of anterior thigh measurements as predictors is both physiologically and statistically justified. However, as the validation was limited to Caucasian adults aged 50 to 76 years, further studies are needed in other ethnic and age groups. The authors also recommended validation in physically active older adults, as 67% of their participants reported regular physical activity at least twice a week [46].

Abe et al. (2018) observed that muscle thickness, along with other variables such as height, age, and sex, can be used with accuracy to estimate muscle mass in older Japanese adults. The linear models using muscle thickness at four sites (R^2^ = 0.902, adjusted R^2^ = 0.899) and one site (R^2^ = 0.868, adjusted R^2^ = 0.866) demonstrated high accuracy and stability in estimating appendicular lean mass minus appendicular fat-free adipose tissue. Bootstrapping (n = 1000) indicated minimal optimism, with values of 0.0062 and 0.0036 for the 4-site and 1-site models, respectively. In that study, 36% of men and 51% of women were classified as having low muscle mass when using the four-parameter equation, indicating that the majority of participants with low muscle mass were women. This finding may be explained by the higher body fat content observed in women, considering that connective and non-fat-free adipose tissues are associated with overall body fat [47].

These results highlight the need for population- and ethnicity-specific equations for estimating muscle mass. The equations developed by Abe et al. (2015) [46] are recommended for Caucasian populations, whereas those proposed by Abe et al. (2018) [47] are more appropriate for the Asian population.

Barbosa-Silva et al. (2021) observed stronger correlations for muscle thickness in the upper limbs compared to the lower limbs. The study involved 190 predominantly female, White, middle-class participants. Two equally accurate equations were developed to estimate appendicular muscle mass in Latin American adults. The most accurate appendicular muscle mass prediction model included two body lengths (height and arm length), two circumferences (dominant arm and thigh), and two ultrasound-based muscle thickness measurements, yielding R^2^ = 0.90 with limits of agreement of ±2.36 kg. A simplified model using only the two lengths and muscle thickness measurements showed comparable performance (R^2^ = 0.89; limits ± 2.51 kg). The simplified model may be more suitable for bedridden individuals or those with clinical conditions (e.g., edema) that may compromise the reliability of circumference-based measurements. Both models provided unbiased appendicular muscle mass estimates that closely matched the DXA values (*p* = 0.13 and 0.09), and bootstrap analysis supported their validity. The limitations of this study include the need for external validation of the proposed equations and limited generalizability beyond the Latin American context. Although novel, the analysis of asymmetry may be challenging to apply in routine practice [19].

Takai et al. (2014) found that combining muscle thickness with limb length or height provides a stronger prediction of muscle mass than using muscle thickness alone. The first Equation (R^2^ = 0.9, SEE = 2.5 kg) and second Equation (R^2^ = 0.9, SEE = 2.0 kg) accurately estimated the free-fat mass. The predicted fat-free mass from both equations (44.4 ± 8.9 kg and 44.4 ± 9.0 kg, respectively) showed no significant difference from the DXA-derived values (44.4 ± 9.2 kg) and exhibited no systematic bias. However, the absolute error was significantly lower for the second Equation (1.5 ± 1.3 kg) than for the first Equation (2.0 ± 1.5 kg). Another limitation noted by the authors was that the ultrasound device used did not exclude intermuscular adipose and connective tissues within the muscle compartment. According to the literature, aging is associated with increased intermuscular fat within the muscle compartment, which may lead to an underestimation of actual muscle size and influence the accuracy of predictive equations. If the accuracy of these equations is affected by the extent of intermuscular fat, then prediction errors may be associated with total body fat or age. However, in the present study, neither age nor total body fat was significantly associated with discrepancies between the predicted and DXA-derived fat-free mass values. Thus, for adults aged 50 to 70 years, the influence of age and fat on prediction accuracy appears minimal, although further research is needed in populations with obesity and a broader age range [21].

The equation developed by Yuguchi et al. (2022), which included three independent variables (gastrocnemius muscle thickness, sex, and BMI), demonstrated a high predictive accuracy. The prediction model for the skeletal muscle mass index showed a high explanatory power, with an adjusted R^2^ of 0.80. The Durbin–Watson statistic was 1.65, indicating no significant autocorrelation, and only one participant fell outside the ±3 SD range. The authors suggested that this equation could provide a non-invasive and simple method for estimating muscle mass when more advanced methods, such as DXA, are not available, particularly in individuals with limited mobility, metal implants, pacemakers, or edema. The limitations of this study include the use of BIA as the reference method and the assessments performed in the seated position, which may differ from the protocols used in other studies. However, this equation has not yet been validated in patients with sarcopenia or unstable clinical conditions, such as those with edema or those in intensive care units. Therefore, further studies are essential to confirm its applicability in broader clinical settings [20].

Paris et al. (2017) showed that including sex, age, and anterior arm muscle thickness improved the four-site muscle thickness protocol used to estimate the appendicular muscle mass. The four-site protocol showed a strong association with appendicular lean mass (R^2^ = 0.72), although Bland–Altman analysis revealed wide limits of agreement (±5.67 kg). The addition of anterior upper arm muscle thickness, age, and sex improved the model (R^2^ = 0.91) and reduced the limits of agreement (±3.18 kg). The optimized model effectively identified low lean mass with an AUC of 0.89. Their equation demonstrated a strong capacity to identify individuals with low lean mass, allowing clinicians to stratify patients by muscle condition (low, moderate, or high risk) at the bedside. However, the model was only internally validated, which could introduce variability when applied to other populations. Furthermore, none of the study participants were classified as underweight by BMI, which limits generalizability to undernourished populations. Due to the sample size and the use of only two evaluators for all analyses, caution is warranted when interpreting the reliability data. Finally, since only biceps and quadriceps muscle thicknesses were used in the protocol, muscle atrophy would only be detected in these regions. This is a limitation, as muscle loss may not be uniformly distributed throughout the body [38].

All studies included in this review underscore the need for validation across different age groups and ethnic populations to ensure the applicability of the proposed equations in clinical practice settings.

### 3.3. Ultrasound for Body Fat Assessment in Older Adults

In addition to muscle mass, the assessment of body fat is of paramount importance in older adults. However, studies evaluating body fat percentage () using ultrasound remain scarce, and further research is needed to assess the applicability of this technique in this population.

Some studies conducted on individuals of different age groups (young people and adults) compared the reliability of DXA and ultrasound in estimating body fat. Chandler et al. (2020) evaluated the reliability of B-mode ultrasound for estimating %BF by comparing intra- and inter-rater measurements from two technicians in a sample of recreationally active adults. The %BF was assessed at seven anatomical sites using modified skinfold-based equations. The results demonstrated excellent intra-rater (ICC ≥ 0.99) and inter-rater reliability (ICC = 0.983), with no significant differences within or between raters (*p* > 0.10). Notably, the inter-rater agreement was stronger in females (ICC = 0.992) than in males (ICC = 0.867), with significant discrepancies observed at individual sites among male participants (*p* < 0.05). These findings support the reproducibility of estimates via ultrasound when standardized protocols are followed, although additional training focused on body composition may further enhance consistency, particularly across sex-specific anatomical regions [48].

Buxadé et al. (2017) assessed the reliability and agreement between skinfold calipers and A-mode ultrasound (Renco Lean-Meater Series 12) for measuring subcutaneous adipose tissue in 84 adults. Skinfold measurements showed excellent intra- and inter-rater reliability (ICC > 0.989), while ultrasound showed lower reproducibility (inter-rater ICC = 0.755). Most anatomical sites showed significant differences between the methods (*p* < 0.001), with weak agreement (Lin’s CCC = –0.009 to −0.646). Discrepancies increased with higher subcutaneous adipose tissue values, especially in participants with greater adiposity. Correlations were poor in certain regions, such as the biceps and abdomen. Regression analysis showed limited explanatory power (adjusted R^2^ = 0.62). Overall, the A-mode device is not interchangeable with calipers [49].

Only one study was conducted exclusively on older adults using A-mode ultrasound [50]. The results of a study published by our research group using portable A-mode ultrasound showed that subcutaneous fat thickness had excellent intra- and inter-rater reliability, with ICCs above 0.90. The lowest reliability was observed for deep abdominal fat, with ICCs of 0.90 and 0.87 for evaluators 1 and 2, respectively, and 0.85 in the inter-rater analysis. Muscle thickness measurements of the triceps, biceps, anterior thigh, and calf demonstrated moderate to good reliability, with ICCs ranging from 0.50 to 0.90. This raises concerns about relying exclusively on A-mode ultrasound to assess muscle mass and diagnose sarcopenia in older adults [50].

Table 3 presents the methodologies of studies that developed predictive equations for estimating %BF using B-mode ultrasound.

**Table 3:** Methodological characteristics of studies that developed predictive equations for estimating body fat percentage (%BF) using B-mode ultrasound.

Author (Year)	Ultrasound Type	Reference Method	Sample Size	Population	Ethnicity	Sex	Age (Years)
Gomez-Perez et al., 2021 [23]	B-mode (L12-4, Philips Ultrasound)	DXA	Development: 104	Healthy adults and older adults	Caucasian	M & F	M: 61.46 ± 6.05/F: 59.71 ± 6.33
Thiebaud et al., 2019 [51]	B-mode (Aloka SSD-500)	DXA	Development: 276/Cross-validation: 138	Healthy adults and older adults	Asian	M & F	50–79

Note. DXA = Dual-energy X-ray absorptiometry; M = male; F = female.

Table 4 presents the predictive equations developed to estimate %BF.

**Table 4:** Predictive equations were developed to estimate body fat percentage (%BF) using B-mode ultrasound.

Author (Year)	Equation Outcome	No. of Parameters	Equation	SEE (%)	R^2^	Adjusted R^2^
Gomez-Perez et al., 2021 [23]	%BF	2	For men: 6.19 + (0.59 × BMI) + (3.26 × ASFT)	2.0	0.8	0.79
		2	For women: 19.16 + (0.74 × BMI) + (0.50 × ASFT)	1.9	0.7	0.71
Thiebaud et al., 2019 [51]	%BF	7	%BF = 15.709 + (1.753 × anterior trunk SFT) + (5.626 × sex [1 = M; 2 = F]) + (3.635 × posterior upper arm SFT) − (4.428 × anterior lower leg SFT) − (0.170 × height) + (0.264 × WC) + (2.241 × anterior thigh SFT)	3.3	0.8	0.80

Note. %BF = Body fat percentage; SFT = subcutaneous fat thickness; ASFT = Abdominal subcutaneous fat thickness; WC = Waist circumference; BMI = Body mass index; Sex: 1 = male; 2 = female.

Gomez-Perez et al. (2021) found that ultrasound-assessed subcutaneous adipose tissue showed strong correlations with total fat mass (females: r = 0.74; males: r = 0.87) and trunk fat mass (females: r = 0.81; males: r = 0.83). Superficial and deep subcutaneous adipose tissues were also significantly correlated (females: r = 0.65 and 0.66; males: r = 0.63 and 0.85; all *p* < 0.0001). Bland–Altman analysis indicated a strong agreement for total and trunk fat mass in both sexes. While estimates showed acceptable agreement in males, a significant proportional bias was observed in females, with a negative slope indicating underestimation (r = –0.298, *p* = 0.0177). The limitations of this study include the lack of external validation in a separate cohort, meaning that the results should be interpreted as preliminary and hypothesis-generating. The authors reported that they were conducting a validation study for the proposed equations. Additionally, the abdominal ultrasound marker used at the umbilicus may be unreliable in individuals with obesity; this limitation was evidenced by the exclusion of two participants from the analysis. Most participants in the study were older women (61%) with obesity (50%) drawn from two major U.S. ethnic groups. Therefore, the generalizability of these equations to other populations remains uncertain. The study also did not provide age-specific data beyond the mean age and did not distinguish between adults and older adults in the analysis [23].

Thiebaud et al. (2019) observed that ultrasound may be used to estimate %BF through subcutaneous fat thickness measurements in middle-aged and older adults. However, they noted significant individual variability between the predicted and DXA-derived %BF values, with evidence of systematic error. Prediction equations for the arm and leg fat mass yielded R^2^ values between 0.690 and 0.812, with SEEs ranging from 0.29 to 0.75 kg. Although the mean bias for estimation was minimal (–0.14%), wide limits of agreement (–8.0 to −7.7%) and significant systematic errors were observed across all equations (r = 0.275 to 0.515, *p* < 0.05). Therefore, caution is recommended when applying these equations to different populations. The authors also noted that it was unclear which variables (anthropometric or ultrasound-based) contributed most significantly to the prediction models, but gradual linear regression was considered appropriate for their analysis. Circumferences of the arm and thigh were not included, although they could potentially improve fat estimates in these regions. Future studies should explore the potential of ultrasound for estimating android and gynoid fat depots [51].

Importantly, all studies included in this review were conducted on ethnically distinct populations. Ethnicity has been shown to influence body composition [52,53], and in Brazil, where the population is highly mixed [54], locally conducted studies are needed to ensure applicability.

Table 5 highlights the advantages and disadvantages of the various equations from our perspective.

**Table 5:** Advantages and disadvantages of various equations from our perspective.

Study (Year)	Advantages	Disadvantages
Abe et al. (2015) [46]	High correlation with DXA; non-invasive.	Limited ethnic diversity.
Abe et al. (2018) [47]	High correlation with DXA; non-invasive.	Limited ethnic diversity.
Barbosa-Silva et al. (2021) [19]	Simple, practical equation.	US measurements were collected and evaluated by a single evaluator.
Takai et al. (2014) [21]	Easy to apply.	Individuals with a BMI below 30 kg/m^2^ participated. Studies on people with obesity are needed.
Paris et al. (2017) [38]	Clinically viable.	Applicability is limited for people with low lean mass; two fixed raters to carry out the analyses.
Yuguchi et al. (2022) [20]	Quick estimate of skeletal muscle index.	Population-specific; lacks DXA comparison.
Thiebaud et al. (2019) [51]	Direct fat measurement; non-invasive.	Plos limits of agreement between the equations and DXA may not be accurate in a clinical setting.
Gomez-Perez et al. (2021) [23]	Good agreement; suitable for bedside use.	%BF: Women had a high degree of bias.

Note. DXA, Dual-energy X-ray absorptiometry; BMI, body mass index; %BF = body fat percentage.

To successfully integrate ultrasound into clinical practice for assessing body composition in older adults, appropriate training and contextual application are essential. Clinicians should receive structured training focused on anatomical landmark identification, probe positioning, and standardized measurement techniques [55,56].

## 4. Conclusions and Future Directions

This narrative review concludes that no studies have used A-mode ultrasound devices to estimate body composition in older adults. Furthermore, there are currently no established cut-off points in the literature to define low muscle mass or high body fat percentage using B-mode ultrasound measurements in older individuals of diverse ethnic backgrounds. Therefore, further studies are necessary to clarify the applicability of ultrasound and the types of equipment used for body composition assessment. At this time, A-mode ultrasound data may serve primarily for monitoring purposes but not for diagnostic use.

Additionally, standardized protocols for body composition assessment using ultrasound are lacking. The measurement accuracy is sensitive to subcutaneous fat thickness, probe angle, and tissue compression. Ultrasound equipment is still predominantly used in hospital settings [16], and image interpretation requires prior training and practice.

The characteristics of each type of equipment should be carefully considered when selecting tools for body composition assessment, including size, cost, portability, frequency (MHz), usability, need for technical training, and whether the device has been validated as a body-composition assessment tool. Higher-frequency transducers provide better image resolution [57]. All the studies reviewed demonstrated that using and interpreting images from B-mode ultrasound requires training and a certain level of expertise. Notably, none of these studies examined A-mode devices, which have very different features from the B-mode and should not be considered equivalent.

Moreover, it is crucial to exercise caution when selecting equations for estimating body composition using ultrasound in clinical practice. Various factors discussed throughout this review—such as the target population, age group, and ethnicity—must be considered. It is well-established that sociodemographic and anthropometric characteristics can influence muscle mass [58] and body fat values.

The applicability of ultrasound-derived equations for estimating body composition in older adults remains limited due to variability in protocols, measurement sites, and population characteristics. While some equations show strong correlations with DXA, their validity across diverse geriatric populations and clinical settings is not yet well established, underscoring the need for broader cross-validation [59].

Cross-validation of ultrasound-based equations for estimating body composition is essential to ensure external validity and applicability across diverse older populations. Many models are developed in specific populations, often homogeneous in age, sex, or ethnicity, which limits their generalizability. Without cross-validation, there is an increased risk of overfitting, where models perform well in the original dataset but poorly in independent clinical datasets. Furthermore, the lack of robust external validation may compromise the clinical decision-making process. Evaluating models across different functional, nutritional, and health statuses enhances generalizability and clinical relevance. Thus, future research should prioritize testing existing equations in independent samples to strengthen the evidence for the use of ultrasound in routine assessments [23].

Ultrasound-based equations for estimating body composition can help support the early identification and monitoring of age-related conditions, such as sarcopenia, enabling personalized nutritional and fitness interventions.

The practical implementation of ultrasound in geriatric care extends beyond its technical validity and requires consideration of real-world barriers and facilitators. A primary limitation is the need for adequate training to ensure standardized image acquisition and interpretation by providers. However, its portability, safety due to non-ionizing radiation, and relatively low cost compared to reference methods such as DXA make ultrasound a promising tool for routine use in bedside and community-based assessments. These characteristics position ultrasound as a feasible and scalable option for geriatric health services, especially in low-resource or decentralized healthcare settings.

## Data Availability

Not applicable.

## References

[1] Gába A., Přidalová M. (2014). Age-Related Changes in Body Composition in a Sample of Czech Women Aged 18-89 Years: A Cross-Sectional Study. Eur. J. Nutr..

[2] Seino S., Shinkai S., Iijima K., Obuchi S., Fujiwara Y., Yoshida H., Kawai H., Nishi M., Murayama H., Taniguchi Y. (2015). Reference Values and Age Differences in Body Composition of Community-Dwelling Older Japanese Men and Women: A Pooled Analysis of Four Cohort Studies. PLoS ONE.

[3] Prado C.M.M., Siervo M., Mire E., Heymsfield S.B., Stephan B.C.M., Broyles S., Smith S.R., Wells J.C.K., Katzmarzyk P.T. (2014). A Population-Based Approach to Define Body-Composition Phenotypes. Am. J. Clin. Nutr..

[4] Mitchell W.K., Williams J., Atherton P., Larvin M., Lund J., Narici M. (2012). Sarcopenia, Dynapenia, and the Impact of Advancing Age on Human Skeletal Muscle Size and Strength; a Quantitative Review. Front. Physiol..

[5] Fernandes L.V., Paiva A.E.G., Silva A.C.B., de Castro I.C., Santiago A.F., de Oliveira E.P., Porto L.C.J. (2021). Prevalence of Sarcopenia According to EWGSOP1 and EWGSOP2 in Older Adults and Their Associations with Unfavorable Health Outcomes: A Systematic Review. Aging Clin. Exp. Res..

[6] Tieland M., Trouwborst I., Clark B.C. (2018). Skeletal Muscle Performance and Ageing. J. Cachexia Sarcopenia Muscle.

[7] Tagliafico A.S., Bignotti B., Torri L., Rossi F. (2022). Sarcopenia: How to Measure, When and Why. Radiol. Medica.

[8] Spahillari A., Mukamal K.J., DeFilippi C., Kizer J.R., Gottdiener J.S., Djoussé L., Lyles M.F., Bartz T.M., Murthy V.L., Shah R.V. (2016). The Association of Lean and Fat Mass with All-Cause Mortality in Older Adults: The Cardiovascular Health Study. Nutr. Metab. Cardiovasc. Dis..

[9] Wang Y., Luo D., Liu J., Song Y., Jiang B., Jiang H. (2023). Low Skeletal Muscle Mass Index and All-Cause Mortality Risk in Adults: A Systematic Review and Meta-Analysis of Prospective Cohort Studies. PLoS ONE.

[10] Varghese M., Song J., Singer K. (2021). Age and Sex: Impact on Adipose Tissue Metabolism and Inflammation. Mech. Ageing Dev..

[11] Kim S., Won C.W. (2022). Sex-Different Changes of Body Composition in Aging: A Systemic Review. Arch. Gerontol. Geriatr..

[12] Rubino F., Cummings D.E., Eckel R.H., Cohen R.V., Wilding J.P.H., Brown W.A., Stanford F.C., Batterham R.L., Farooqi I.S., Farpour-Lambert N.J. (2025). Definition and Diagnostic Criteria of Clinical Obesity. Lancet Diabetes Endocrinol..

[13] Newman A.B., Visser M., Kritchevsky S.B., Simonsick E., Cawthon P.M., Harris T.B. (2023). The Health, Aging, and Body Composition (Health ABC) Study-Ground-Breaking Science for 25 Years and Counting. J. Gerontol. Ser. A Biol. Sci. Med. Sci..

[14] Prado C.M., Ford K.L., Gonzalez M.C., Murnane L.C., Gillis C., Wischmeyer P.E., Morrison C.A., Lobo D.N. (2023). Nascent to Novel Methods to Evaluate Malnutrition and Frailty in the Surgical Patient. J. Parenter. Enter. Nutr..

[15] Price K.L., Earthman C.P. (2019). Update on Body Composition Tools in Clinical Settings: Computed Tomography, Ultrasound, and Bioimpedance Applications for Assessment and Monitoring. Eur. J. Clin. Nutr..

[16] Prado C.M., Landi F., Chew S.T.H., Atherton P.J., Molinger J., Ruck T., Gonzalez M.C. (2022). Advances in Muscle Health and Nutrition: A Toolkit for Healthcare Professionals. Clin. Nutr..

[17] Cruz-Jentoft A.J., Bahat G.G., Bauer J.J., Boirie Y., Bruyere O., Cederholm T., Cooper C., Landi F., Rolland Y., Sayer A.A. (2019). Sarcopenia: Revised European Consensus on Definition and Diagnosis. Age Ageing.

[18] Jeon S., Kim M., Yoon J., Lee S., Youm S. (2023). Machine Learning-Based Obesity Classification Considering 3D Body Scanner Measurements. Sci. Rep..

[19] Barbosa-Silva T.G., Gonzalez M.C., Bielemann R.M., Santos L.P., dos Costa C.S., Menezes A.M.B. (2021). 2 + 2 (+ 2) = 4: A New Approach for Appendicular Muscle Mass Assessment by Ultrasound. Nutrition.

[20] Yuguchi S., Asahi R., Kamo T., Azami M., Ogihara H. (2022). Prediction Model Including Gastrocnemius Thickness for the Skeletal Muscle Mass Index in Japanese Older Adults. Int. J. Environ. Res. Public Health.

[21] Takai Y., Ohta M., Akagi R., Kato E., Wakahara T., Kawakami Y., Fukunaga T., Kanehisa H. (2014). Applicability of Ultrasound Muscle Thickness Measurements for Predicting Fat-Free Mass in Elderly Population. J. Nutr. Heal. Aging.

[22] Yeung S.S.Y., Reijnierse E.M., Pham V.K., Trappenburg M.C., Lim W.K., Meskers C.G.M., Maier A.B. (2019). Sarcopenia and Its Association with Falls and Fractures in Older Adults: A Systematic Review and Meta-Analysis. J. Cachexia Sarcopenia Muscle.

[23] Gomez-Perez S.L., Zhang Y., Mourtzakis M., Tussing-Humphreys L., Ridlon J., Gaskins H.R., Mutlu E. (2021). Comparison between Handheld Ultrasound and Regional and Whole-Body Dual Energy x-Ray Absorptiometry (DXA) for Body Fat Assessment. Clin. Nutr. ESPEN.

[24] Van den Broeck J., Buzzatti L., Jager-Wittenaar H., Perkisas S., Scafoglieri A. (2021). The Validity of Ultrasound-Derived Equation Models to Predict Whole-Body Muscle Mass: A Systematic Review. Clin. Nutr. ESPEN.

[25] Kokura Y., Nishioka S., Maeda K., Wakabayashi H. (2023). Ultrasound Utilized by Registered Dietitians for Body Composition Measurement, Nutritional Assessment, and Nutritional Management. Clin. Nutr. ESPEN.

[26] Akazawa N., Kishi M., Hino T., Tsuji R., Tamura K., Moriyama H. (2021). Using GLIM Criteria, Cutoff Value for Low BMI in Asian Populations Discriminates High or Low Muscle Mass: A Cross-Sectional Study. Nutrition.

[27] Rodrigues C.N., Ribeiro Henrique J., Ferreira Á.R.S., Correia M.I.T.D. (2021). Ultrasonography and Other Nutrition Assessment Methods to Monitor the Nutrition Status of Critically Ill Patients. J. Parenter. Enter. Nutr..

[28] Van Ruijven I.M., Stapel S.N., Molinger J., Weijs P.J.M. (2021). Monitoring Muscle Mass Using Ultrasound: A Key Role in Critical Care. Curr. Opin. Crit. Care.

[29] Jahreis T., Kretschmann J., Weidner N., Volk T., Meiser A., Groesdonk H.V. (2021). Sonographic Evaluation of Gastric Residual Volume during Enteral Nutrition in Critically Ill Patients Using a Miniaturized Ultrasound Device. J. Clin. Med..

[30] Cederholm T., Jensen G.L., Correia M.I.T.D., Gonzalez M.C., Fukushima R., Higashiguchi T., Baptista G., Barazzoni R., Blaauw R., Coats A. (2019). GLIM Criteria for the Diagnosis of Malnutrition—A Consensus Report from the Global Clinical Nutrition Community. Clin. Nutr..

[31] De Nutricionistas-CFN, Conselho Federal (2023). Uso de Ultrassonografia Para Avaliação de Composição Corporal Pelo Nutricionista. CFN—Nota Técnica No. 83/2023..

[32] Nijholt W., Scafoglieri A., Jager-Wittenaar H., Hobbelen J.S.M., van der Schans C.P. (2017). The Reliability and Validity of Ultrasound to Quantify Muscles in Older Adults: A Systematic Review. J. Cachexia Sarcopenia Muscle.

[33] Ferreira L.F., da Silva E.B., Bomfim A.B.C. (2024). Validity and Reliability of Portable A-Mode Ultrasound in Measuring Body Fat Percentage: A Systematic Review with Meta-Analysis. PLoS ONE.

[34] Zhao R., Li X., Jiang Y., Su N., Li J., Kang L., Zhang Y., Yang M. (2022). Evaluation of Appendicular Muscle Mass in Sarcopenia in Older Adults Using Ultrasonography: A Systematic Review and Meta-Analysis. Gerontology.

[35] Baethge C., Goldbeck-Wood S., Mertens S. (2019). SANRA Una Escala Para La Evaluación de La Calidad de Los Artículos de Revisión Narrativa. [SANRA—A Scale for the Quality Assessment of Narrative Review Articles]. Res. Integr. Peer Rev..

[36] Holmes C.J., Racette S.B. (2021). The Utility of Body Composition Assessment in Nutrition and Clinical Practice: An Overview of Current Methodology. Nutrients.

[37] Wagner D.R., Teramoto M., Judd T., Gordon J., McPherson C., Robison A. (2020). Comparison of A-Mode and B-Mode Ultrasound for Measurement of Subcutaneous Fat. Ultrasound Med. Biol..

[38] Paris M.T., Lafleur B., Dubin J.A., Mourtzakis M. (2017). Development of a Bedside Viable Ultrasound Protocol to Quantify Appendicular Lean Tissue Mass. J. Cachexia Sarcopenia Muscle.

[39] Tang X., Huang L., Yue J., Qiu L. (2022). Quantitative Estimation of Muscle Mass in Older Adults at Risk of Sarcopenia Using Ultrasound: A Cross-Sectional Study. Quant. Imaging Med. Surg..

[40] Ido A., Nakayama Y., Ishii K., Iemitsu M., Sato K., Fujimoto M., Kurihara T., Hamaoka T., Satoh-Asahara N., Sanada K. (2015). Ultrasound-Derived Abdominal Muscle Thickness Better Detects Metabolic Syndrome Risk in Obese Patients than Skeletal Muscle Index Measured by Dual-Energy X-Ray Absorptiometry. PLoS ONE.

[41] Fukumoto Y., Ikezoe T., Taniguchi M., Yamada Y., Sawano S., Minani S., Asai T., Kimura M., Ichihashi N. (2021). Cut-off Values for Lower Limb Muscle Thickness to Detect Low Muscle Mass for Sarcopenia in Older Adults. Clin. Interv. Aging.

[42] Abe T., Loenneke J.P., Thiebaud R.S. (2018). The Use of Ultrasound for the Estimation of Muscle Mass: One Site Fits Most?. J. Cachexia Sarcopenia Muscle.

[43] Lunet N., Severo M., Barros H. (2006). Desvio Padrão Ou Erro Padrão. Arq. Med..

[44] Lohman T.G. (1992). Advances in Body Composition Assessment.

[45] Dodge Y. (2008). Coefficient of Determination. The Concise Encyclopedia of Statistics.

[46] Abe T., Thiebaud R.S., Loenneke J.P., Young K.C. (2015). Prediction and Validation of DXA-Derived Appendicular Lean Soft Tissue Mass by Ultrasound in Older Adults. Age.

[47] Abe T., Thiebaud R.S., Loenneke J.P., Fujita E., Akamine T. (2018). DXA-Rectified Appendicular Lean Mass: Development of Ultrasound Prediction Models in Older Adults. J. Nutr. Heal. Aging.

[48] Chandler A.J., Dona S.T., Cintineo H.P., Mcfadden B.A., Sanders D.J., Monaco R., Arent S.M. (2020). Intra- and Inter-Rater Reliability of Assessing Body Composition Using B-Mode Ultrasound in Conjunction with Artificial Intelligence Software. J. Exerc. Nutr..

[49] Buxadé C.P.C., Solà-Perez T., Castizo-Olier J., Carrasco-Marginet M., Roy A., Marfell-Jones M., Irurtia A. (2018). Assessing Subcutaneous Adipose Tissue by Simple and Portable Field Instruments: Skinfolds versus A-Mode Ultrasound Measurements. PLoS ONE.

[50] De Oliveira G.B., Fernandes L.V., Chen X.S., Andrade F.C.D., Costa L.S., Vasques A.C.J., Corona L.P. (2024). Intra- and Inter-Rater Reliability of Muscle and Fat Thickness Measurements Obtained Using Portable Ultrasonography in Older Adults. Clin. Nutr. ESPEN.

[51] Thiebaud R.S., Abe T., Loenneke J.P., Fujita E., Akamine T. (2019). Body Fat Percentage Assessment by Ultrasound Subcutaneous Fat Thickness Measurements in Middle-Aged and Older Adults. Clin. Nutr..

[52] Jeong S.M., Lee D.H., Rezende L.F.M., Giovannucci E.L. (2023). Different Correlation of Body Mass Index with Body Fatness and Obesity-Related Biomarker According to Age, Sex and Race-Ethnicity. Sci. Rep..

[53] Heymsfield S.B., Peterson C.M., Thomas D.M., Heo M., Schuna J.M. (2016). Why Are There Race/Ethnic Differences in Adult Body Mass Index–Adiposity Relationships? A Quantitative Critical Review. Obes. Rev..

[54] Callegari-Jacques S.M., Grattapaglia D., Salzano F.M., Salamoni S.P., Crossetti S.G., Ferreira M.E., Hutz M.H. (2003). Historical Genetics: Spatiotemporal Analysis of the Formation of the Brazilian Population. Am. J. Hum. Biol..

[55] Miclos-Balica M., Muntean P., Schick F., Haragus H.G., Glisici B., Pupazan V., Neagu A., Neagu M. (2020). Reliability of Body Composition Assessment Using A-Mode Ultrasound in a Heterogeneous Sample. Eur. J. Clin. Nutr..

[56] Müller W., Lohman T.G., Stewart A.D., Maughan R.J., Meyer N.L., Sardinha L.B., Kirihennedige N., Reguant-Closa A., Risoul-Salas V., Sundgot-Borgen J. (2016). Subcutaneous Fat Patterning in Athletes: Selection of Appropriate Sites and Standardisation of a Novel Ultrasound Measurement Technique: Ad Hoc Working Group on Body Composition, Health and Performance, under the Auspices of the IOC Medical Commission. Br. J. Sports Med..

[57] Wagner D.R. (2013). Ultrasound as a Tool to Assess Body Fat. J. Obes..

[58] Nagae M., Umegaki H., Yoshiko A., Fujita K. (2023). Muscle Ultrasound and Its Application to Point-of-Care Ultrasonography: A Narrative Review. Ann. Med..

[59] Ticinesi A., Meschi T., Narici M.V., Lauretani F., Maggio M. (2017). Muscle Ultrasound and Sarcopenia in Older Individuals: A Clinical Perspective. J. Am. Med. Dir. Assoc..

